# Challenges to Oil Spill Assessment for Seabirds in the Deep Ocean

**DOI:** 10.1007/s00244-016-0355-8

**Published:** 2017-07-10

**Authors:** J. Christopher Haney, Patrick G. R. Jodice, William A. Montevecchi, David C. Evers

**Affiliations:** 1 Terra Mar Applied Sciences, LLC, 123 W. Nye Lane, Suite 129, Carson City, NV 89706 USA; 2 U.S. Geological Survey South Carolina Cooperative Fish and Wildlife Research Unit and Department of Forestry and Environmental Conservation, Clemson University, Clemson, SC 29634 USA; 3 Memorial University of Newfoundland, St. John’s, Newfoundland and Labrador A1B 3X9 Canada; 4 Biodiversity Research Institute, 276 Canco Road, Portland, ME 04103 USA

## Abstract

We synthesize impediments for evaluating effects to seabirds from open ocean hydrocarbon releases. Effects on seabirds from ship discharges, spills, and well blowouts often are poorly detected and monitored far from land. Regulatory regimes for ocean spills can result in monitoring efforts that are not entirely transparent. We illustrate how interdisciplinary technologies address deficits that hamper individual or population level assessments for seabirds, and we demonstrate where emerging technologies might be engaged to bridge gaps in oil spill monitoring. Although acute mortality from direct oil exposure poses the greatest risk to seabirds, other hazards from light-attraction, flaring, collisions, chronic pollution, and hydrocarbon inhalation around oil infrastructure also may induce bird mortality in the deep ocean.

Throughout the world’s oceans, seabirds are sensitive and convenient tracers of hydrocarbon contamination. Marine birds use diverse foraging styles, contact sea water frequently, and adhere oil to plumage. Seabirds integrate environmental information (Parsons et al. 2008) as sensors for external and internal exposure (Leighton 1993). Petroleum damages feather microstructure (O’Hara and Morandin 2010) and compromises avian health, leading to morbidity or mortality from dehydration, weight loss, decreased hematological functioning, poisoning, drowning, hypothermia, and/or starvation (Jenssen 1994; Briggs et al. 1996; Paruk et al. 2016).

Enumerating how seabirds are affected by marine oil spills is complicated enough near shore (Piatt et al. 1990; Castege et al. 2007). Assessing impacts for spills in deep ocean environments is more challenging (Haney et al. 2014a). Yet, exploration and drilling continue to increase in deep-sea environments (Pettingill and Weimer 2002). Small, transient discharges as well as extensive, protracted blowouts have occurred in remote deep-sea regions of both low arctic (Wilhelm et al. 2007) and warm tropical oceans (Brushett et al. 2011). Even small oil spills result in mortality to tens of thousands of birds (Barrett 1979; Burger 1993).

We provide spill responders with a primer on the challenges to evaluate acute impacts of oil spills on marine birds at individual and population levels. We emphasize frontiers where a need for assessment and technological innovation are most essential. We furnish examples that illustrate how impact assessments for marine birds can be improved. Our review stresses direct, acute impacts only, although we recognize that chronic effects from spills may affect birds and their environments over years or decades (Peterson et al. 2003; Fraser and Racine 2016).

## Regulatory Policies: Effectiveness, Conflicts, Examples and Lessons

### Regulatory Policies

Deep ocean spills usually carry obligations that polluters compensate for environmental damages to marine biota, such as seabirds, although application of these rules is not always consistent or effective (Caballero-Miguez and Fernández-González 2015). In the United States, assessing injury from oil spills on wildlife includes monetization of the mortality (Migratory Bird Treaty Act) or the lost uses of injured resources and services (Oil Pollution Act; Sperduto et al. 2003). Prerequisites for environmental assessment and compensation for damages caused by oil spills, however, vary greatly across national jurisdictions. Consequently, approaches to spill assessment can vary substantially (Liu and Zhu 2014; Mendes et al. 2014).

Primary needs for assessment require independent observation and transparent information about onsite operations and wildlife interactions (Burke et al. 2012). Otherwise, operators may misreport spills and discharges, the magnitude of affected seabirds, and other key factors for quantifying impacts. Amplified by lack of compliance with environmental response plans (Fraser and Racine 2016), these circumstances create scenarios where missing information can be interpreted falsely as indicative of no problem—analogous to Type II statistical errors in environmental assessments (Buhl-Mortensen 1996).

### Policy Context

In the United States, the Oil Pollution Act of 1990 created a federal program, the Natural Resource Damage Assessment and Restoration (NRDAR), for assessing and ultimately restoring any injuries to fish, wildlife, and other natural resources. The Departments of Interior and Commerce, along with state, tribal, and other federal partners, act as resource “Trustees.” Trustees identify the type and extent of natural resources injured, recover restoration costs from those responsible, and then plan and perform long-term restoration activities.

In Canada, regulatory policies in Canadian Atlantic Accord (Section 119) provide operators with confidentiality regarding spill response plans and spill reporting. To illustrate, the Canadian Commissioner of the Environment highlighted “oil and gas exploration and drilling activities are exempt from reporting pollutant releases to Environment Canada” (Office of the Auditor General of Canada 2012). This approach has weakened environmental validation and mitigation (Fraser and Racine 2016). For example, on May 10, 2010, within a few weeks of the ongoing *Deepwater Horizon* (DWH) blowout, the *Trans*-*Ocean Stena Carron* drilled the deepest offshore well (2.5 km) in Canadian history in the Orphan Basin on the Grand Banks. Despite a flotilla of vessels assisting in the exercise, no independent environmental or seabird observers were present. The report of a recent public hearing on offshore oil development indicates that although hydrocarbon platforms have been operating on the eastern Canadian shelf for two decades, “A review of the previous intervention by environmentalists has revealed that the same outstanding questions remain unanswered after 20 years of public hearings” (Canada-Newfoundland Offshore Petroleum Board 2012).

Even when events are reported, limitations occur. The 2004 offshore spill of an operator-estimated 1000-barrel (159,000 L) spill of crude oil at the *Terra Nova* Floating Platform (Grand Banks Canada) involved initial reports of no interactions with seabirds. These reports were repeated by the regulator—the Canada-Newfoundland and Labrador Offshore Petroleum Board (C-NLOPB). Yet, a subsequent exposure probability model using satellite imagery and vessel surveys through the slick area 1 week after the spill (and after spill dissipation) estimated that 10,000 murres (*Uria* spp.) and dovekies (*Alle alle*) were killed (Wilhelm et al. 2007). This exercise was precedent-setting in that no direct data were available on seabird mortality (also see below).

## Exposure to Individual Birds

### Hydrocarbon Detection on Carcasses and Live Birds

Detection and quantification of oiling rates on seabirds can be conducted remotely (e.g., through observation and photography) and in-hand (e.g., through blood physiology and external ultraviolet (UV) fluorescence). In the past, estimating the number of oiled seabirds through carcass retrieval was the primary means to assess injury (Ford 2006; Ford and Zafonte 2009). In association with the DWH blowout, efforts were made to expand and refine the process of assessing injury to include not only heavily oiled birds that were beached or otherwise severely compromised but also lightly to moderately oiled birds that were still mobile.

For the DWH, researchers collected data on oiling rates of free-ranging marine birds (in flight or on land or water) within a set gridded or transect system over an established time period and evaluated birds that had more than 50% of their body in clear view of the observer. Surveys were conducted from boat and land generally within 80 m of focal individuals. Boats were anchored or moved slowly and observers used 10 × 42 binoculars. Observations also were recorded with a digital camera with a 100- to 400-mm lens. Because they were replicable and reviewable, photographs proved to be a more definitive method to estimate oiling levels and rates in birds. Standard oiling levels for the DWH were categorized according to oil coverage for each bird: 1) not visibly oiled, 2) trace (<5%), 3) light (5–20%), 4) moderate (20–40%), 5) heavy (>40%) (Evers et al. 2011).

New field assessments were piloted during the DWH to assess impacts of oil on northern gannet *Morus bassanus*, brown pelican *Pelecanus occidentalis*, and common loon *Gavia immer*. For each species, blood was collected to evaluate health parameters and PAH levels and subsequently related to physical measurements, such as body mass (Paruk et al. 2016). In hand, birds also were evaluated for the presence and location of trace or light oiling using UV fluorescence (Paruk et al. 2016). Although still exploratory, UV fluorescence has the potential to refine injury assessment further as preliminary results suggest that physiological changes in oiled birds might be correlated with levels of UV fluorescence.

### Hydrocarbon Risk Assessment using Tracking Information

Seabirds range widely and can encounter offshore spills over an extensive area. Deployment of tracking devices provides a tool with which spatial links between spill sites and use areas of individuals, both on land and at sea, can be assessed over large, distant expanses (1000 s of km^2^) for extended periods (years). At any time of year, offshore areas support breeding and migrating seabirds. As such the effective reach of the spill can extend to nearby breeding sites (e.g., nearshore species with foraging ranges on the order of 10 s of km; Lamb 2016) or to very distant breeding colonies (e.g., migrants that can be 1000 s of km from breeding sites; Montevecchi et al. 2012). Tracking data also provide a means to model habitat use and residency time within an area (Wakefield et al. 2009) and hence predict exposure risk to species, a valuable tool during the early stages of spill response.

Tracking also informs spatial aspects of exposure that might otherwise be overlooked or misinterpreted. For example, tracking data often extend the documented range of seabirds beyond that estimated from at-sea observations. Jodice et al. (2015) demonstrated that satellite-tagged black-capped petrels *Pterodroma hasitata* ranged farther east of the Atlantic Gulf Stream than previously documented from ship-based surveys. Similarly, Fifield et al. (2014) demonstrated that a component of the northern gannet population breeding in Newfoundland made trans-Atlantic migrations and wintered off the coast of West Africa. Although neither example assessed spill exposure per se, both demonstrate the extent to which tracking elucidates a species’ range and ultimately its overall exposure risk.

Tracking data also can document intra- and inter-specific differences in survival rates following a spill. Evers et al. (2011) used satellite tags to monitor daily survival of two species of nearshore seabird with different life-history strategies in oiled and unoiled ecosystems following the DWH. Individual-based data, such as sex, body condition, and visible oiling levels, were included as covariates for survival assessment. Satellite tags also can provide location data at regular intervals, which can provide detailed foraging and migration ranges. Before the DWH, there were no explicit data on foraging ranges or migratory patterns of brown pelicans in the Gulf of Mexico, and few from anywhere in the species’ ranges, confounding understanding of exposure risk. Recently, however, Lamb (2016) used GPS satellite tags and showed that, across six colonies in the northern Gulf, the foraging distances (10–100 km) of breeding adults and migratory patterns ranged widely (100 to >2000 km). Such variability suggests that a “one-size fits all” approach is inappropriate when assessing risk to a species even within a single region. Location data collected at regular intervals also can be used to estimate travel speed and applied to exposure probability models, particularly for rare species not readily observed during surveys or collected as carcasses.

Despite their growing accessibility and affordability, deployment of tracking devices on seabirds requires considerable expertise. Mass of tracking devices is recommended at ≤3% of body mass of the target species to minimize negative effects of payload (Vandenabeele et al. 2012), and shape and placement of the tag should be designed to minimize aero- and hydrodynamic drag. Attachment techniques are taxa dependent and affect attachment longevity. Tags taped to feathers can last days to months (Patrick and Weimerskirch 2014), tags sutured on can last weeks to months (Pollett et al. 2014), and tags implanted or secured with backpack harness or leg bands can last years (De La Cruz et al. 2013). Devices have distinct spatial and temporal resolutions related to size, mass, software and power source. Platform Terminal Transmitters (PTTs, or satellite tags) are typically accurate to 500–1500 m and can provide several locations per day for multiple years. GPS technology, whether paired with a PTT or cellular transmission terminal (CTT) that actively transmit data or deployed as an archival device, can provide location accuracy of <10 m and many locations daily. Duration can last days to years depending upon the device. Geolocating light sensors (GLS or geolocators) provide one or two locations per day at a very coarse resolution (ca. 150–200 km) for one to several years. GLS are archival and hence require retrieval but can be lightweight (≤2 g) and often are the only viable choice for small seabirds (<200 g). Size and mass of GPS, however, are trending towards miniaturization and therefore become more suitable for a wider range of species. Costs vary from ~US $100–$4000 among all of the aforementioned devices.

## Exposure at Population Levels

### Carcass Sampling Models

Until a decade ago, the only method readily used to estimate population-level mortality from a spill was to tally the location, number, and composition of carcasses. This indirect method relied upon extrapolations from recoveries to estimate more realistic totals of birds killed (Piatt et al. 1990). Recovering all birds incapacitated or killed by an oil spill is impossible, particularly when the spill occurs in pelagic waters. For example, although carcass sampling identified mortality at the species level following the DWH spill, the warm-water pelagic environment of the Gulf of Mexico made it difficult to obtain accurate estimates of individual-level mortality (Haney et al. 2014a)

Inflation factors allow for carcasses lost, missed, or otherwise unobservable. Inflation factors address sinking from loss of buoyancy, wind and current speed and direction, proportion of beach area searched, undetected carcasses, and carcasses taken by marine and coastal scavengers before being counted. Because each inflation factor carries a parameter value having statistical error, the overall estimate for bird mortality can be magnified and result in wide uncertainty from cumulative error (Haney et al. 2014b).

Field detection, monitoring, and calibration efforts in carcass sampling models are extensive and expensive. Great effort must be expended to regularly search and find as many carcasses as possible, attending to forensic and chain-of-custody protocols, especially if the spill violated regulation (Schoenbaum 2012). Shorelines and other depositional environments must be searched frequently and carcasses retrieved at intervals that minimize scavenger removals (Ford 2006). Likelihood of detection and duration of carcass persistence must be calibrated (Byrd et al. 2009). Drift velocity and direction from the offshore oil slick (Castege et al. 2007), and sinking rates from decomposition or scavenging (Wiese 2003), must be estimated for carcasses that reach shorelines. Adjustments to parameters account for exposure, deposition, or detection rates that vary by carcass body size (Ford and Zafonte 2009).

Carcass sampling models thus require that substantial financial, logistical, and other resources be available and invested to be effective. Consequently, such models may be inaccessible to oversight organizations as well as to the general public.

### Exposure Probability Models

When prevailing currents thwart shoreline carcass deposition, mortality of marine birds can be estimated with an exposure probability model (Wilhelm et al. 2007; Haney et al. 2014a). For a given spill duration, exposure probability models enumerate mortality with as few as three parameters: bird density at sea, proportionate mortality due to oiling, and spatial extent of the oil slick. Mortality is then calculated through multiplication of these three parameters. Values for proportionate mortality and spatial extent of a spill often are available in the public domain (e.g., web portals, published manuscripts), and therefore researchers and the public have a means by which claims made by the polluter or regulatory agency can be verified. However, seabird distribution data often are not as readily available across all marine ecosystems, and those data that are available may be dated or otherwise limited spatially and temporally.

Bird densities may be collected prior to spills, especially in offshore regions where baseline data are required before and during energy development (e.g., the North Sea; Begg et al. 1997). Proportionate mortality due to oiling can be modeled with values ranging between 0 and 100% to represent that fraction of birds exposed lethally. Given poor long-term prospects of birds dosed even with trace oil (Sharp 1996), values for proportionate mortality usually fall near the middle and upper end of this range, 50% or higher (Wilhelm et al. 2007; Haney et al. 2014b). A parameter value for slick area also can be estimated by using satellite imagery that delineates the daily or cumulative slick size or using calculators for spreading rates from spills of known volume and fuel type (MSANZ 2016).

**Fig. 1:**
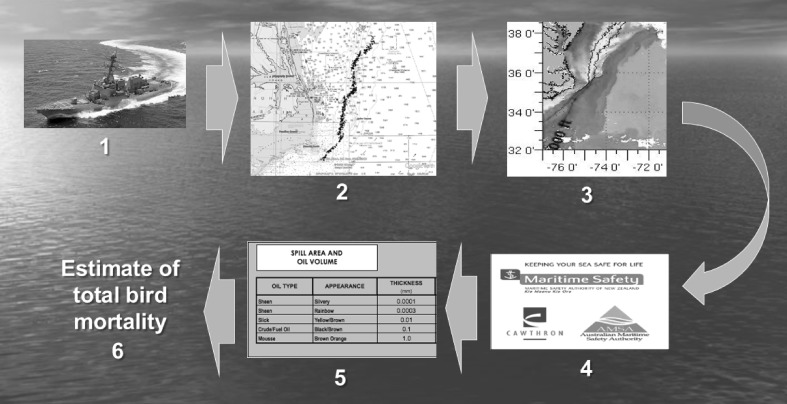
Converting an “invisible,” ephemeral oil spill into marine bird mortality. (1) On March 12, 2014, a naval vessel accidentally discharged military-grade diesel fuel for 7 h off North Carolina’s Outer Banks. (2) Discharge time, distance, volume, and track line given by ship records. (3) Real-time satellite imagery confirmed spill at Gulf Stream edge, limiting cross-shelf transport. (4) Volume-to-spill area calculator projected slick’s spatial extent, mostly sheen given low viscosity in this volatile fuel. (5) Slick narrow and long; size estimated 183 km × 7.3 km = 1336 km^2^. (6) Seabird densities (adjusted for statistical uncertainty) multiplied by spill area estimated 400–2800 birds killed within 24–36 h

Exposure probability models can be applied even to transitory spills far from land where mounting a monitoring response is not feasible. During a 7-h span on March 11–12, 2014, the U.S.S. *Jason Dunham* accidentally released 130,000 L of marine diesel off North Carolina’s Outer Banks. Satellite imagery juxtaposed with the cruise track showed a discharge just inside the western Gulf Stream boundary (Fig. 1). Fuel Oil No. 2 has a high evaporation rate and spreads quickly into thin films of sheen.

Using a calculator to convert spill volume into surface area given this fuel type (MSANZ 2016), slick size for the *Jason Dunham* spill was estimated at 1336 km^2^. With proportionate mortality for birds estimated between 20 and 60%, and seabird densities in this season and location of 1.6–3.5 birds km^−2^, total mortality was estimated at 400–2800 birds (Haney unpublished data). Numbers of birds affected decline if a volatile fuel dissipates rapidly at the sea surface (e.g., from turbulence), thereby curtailing total exposure time.

## Discussion

Coastal groundings by tankers can trigger on the order of 10^4^–10^5^ bird deaths (Piatt et al. 1990; Castege et al. 2007). Tanker spills have declined in number and volume worldwide, however, such that large releases from offshore production rigs (Wilhelm et al. 2007) and huge well blowouts pose some of the highest mortalities in recent years.

The catastrophic blowout of the DWH drill rig lasted 103 days, covered 175,000 km^2^ (Norse and Amos 2010), and killed up to 10^6^ seabirds in coastal and offshore waters (Haney et al. 2014a, b). The 2009 *Montara* (West Atlas) blowout in the Timor Sea also stemmed from loss of well control (but at 76 m vs. 1500 m in DWH). Bird deaths from the *Montara* blowout are unknown, but mortality was likely high given numerous birds over the slick (Watson et al. 2009), a spill duration of 74 days, and oil, wax, and sheen from the blowout covering 95,000 km^2^ (Asia Pacific ASA 2010).

Beached bird surveys, the standard way to assess seabird mortality from contamination (Camhuysen and Huebeck 2001), are compromised if the discharge is from offshore platforms (Robertson et al. 2012). Onsite observers and/or automated sensors on platforms could reduce uncertainty related to seabird attraction to platforms, mortality events, and chronic spills and discharges (Fraser and Racine 2016). Such an approach is excluded, however, by regulatory regimes throughout the world’s oceans (Burke et al. 2012). Furthermore, new research is needed to understand novel risks to seabirds from hydrocarbon contamination. Although not well studied, avian survival rates in warm versus cold seas, respiratory exposure to volatile hydrocarbons at the air-sea interface (Gagnon and Rawson 2010), and behavioral avoidance of or attraction to spills would notably improve mortality modeling.

Bird-borne tracking attachments help identify and quantify exposure risks (Hedd et al. 2011; Montevecchi et al. 2012) and can be informative in transboundary events (Jodice and Suryan 2010). Tracking data extend the footprint of spill impact by linking breeding colonies of exposed birds to distant spill sites (Montevecchi et al. 2012). Individual movement data inform habitat and survival modeling, enhancing our ability to assess spill impact whether at drilling rigs or along oil shipping routes beyond immediate mortality.

Baseline surveys of population density at sea and composition of species vulnerable to exposure are the topmost priorities for spill assessment. Ship surveys can be used to assess habitat use and diversity (Burke et al. 2006) and can be coupled with species movement patterns to model seabird distributions. Distribution and movement patterns of seabirds, along with satellite imagery of spill dimensions, can then reduce uncertainty in the assessment of bird mortality from deep ocean spills (Wilhelm et al. 2007). Ongoing long-term research on the distribution and habitats associations of birds at sea is essential for reducing crisis management when inevitable calamities arise.
